# Factors predicting hip joint aspiration yield or “dry taps” in patients with total hip arthroplasty

**DOI:** 10.1186/s13018-022-02942-8

**Published:** 2022-01-22

**Authors:** Justin Ong, Alex Tang, Joshua C. Rozell, James S. Babb, Ran Schwarzkopf, Dana Lin

**Affiliations:** NYU Langone Health, New York, USA

**Keywords:** Saline lavage, Dry tap, Periprosthetic joint infection, Total hip arthroplasty

## Abstract

**Background:**

Image-guided joint aspirations used to assist the diagnosis of periprosthetic joint infection (PJI) may commonly result in a dry tap–or insufficient fluid for culture and cell count analysis. Dry tap aspirations are painful and invasive for patients and often utilize a subsequent saline lavage to obtain a microbiology sample. Currently, there is a paucity of the literature addressing predictors that could suggest whether a dry tap will occur. The purpose of this study was to examine the effects of various factors on “dry tap” occurrence in patients with suspected PJI following total hip arthroplasty (THA).

**Methods:**

A retrospective review was performed among THA patients suspected for PJI who received image-guided joint aspiration procedures at our institution from May 2016 to February 2020. The procedural factors included the imaging modality used for aspiration, anatomic approach, needle gauge size used, and the presence of a trainee. The patient-specific factors included number of prior ipsilateral hip surgeries, femoral head size, ESR/CRP values, and BMI.

**Results:**

In total, 336 patients met our inclusion criteria. One hundred and twenty hip aspirations resulted in a dry tap (35.7%) where the patients underwent a saline lavage. Among the procedural and patient-specific factors, none of the factors were found to be statistically different between the two cohorts nor conferred any greater odds of a dry tap occurring.

**Conclusion:**

No associations with dry tap occurrence were found among the procedural and patient-specific factors studied. Further research is needed to identify additional factors that may be more predictive of dry taps.

## Background

Synovial fluid aspiration of the hip joint is a commonly performed procedure that plays an important role in the diagnosis of suspected periprosthetic joint infection (PJI) following total hip arthroplasty (THA) [1–4]. In addition to determining synovial white blood cell (WBC) count and neutrophil differential via polymorphonuclear percentage (PMN%), joint aspiration culture can identify the casual organism and antibiotic sensitivities [1, 5]. Infections are commonly sustained by skin contaminating bacteria (i.e., *S. aureus* and *S. epidermidis*) though organisms such as Mycobacterium tuberculosis can be more frequent in low income countries [6]. Findings from the joint aspiration help guide surgical management and determine the most appropriate course of antibiotic treatment for infected patients [1, 7].

However, synovial fluid may not be obtainable at the time of aspiration, resulting in a “dry tap” that can be painful for the patient and may preclude the ability to culture a sample [8, 9]. Previous studies have shown that rates of obtaining a dry tap may occur in as high as 46–49% of cases [10, 11]. These occurrences may also happen more frequently in the hip than in the knee, as one study reported a rate of 41.8% following THA and 15% following total knee arthroplasty [12].

To date, there are no studies that address predictive factors that could suggest whether a dry tap will occur. These factors may be related to technical components related to the joint aspiration such as aspiration imaging modality, anatomic approach, needle gauge size, and the educational environment, such as when the aspiration is performed by the trainee and directly supervised by the attending MSK-trained radiologist. Patient-related factors may also be predictive such as erythrocyte sedimentation rate (ESR) and C-reactive protein (CRP) values, body mass index (BMI), femoral head size, and number of revision surgeries [2, 13–25]. Larger femoral head sizes were hypothesized to result in larger elastohydronamic lubrication and larger surface area, therefore potentially leading to larger fluid volume. Such information would be valuable in aiding MSK-trained radiologists to reliably identify patients at risk for a dry tap and may help improve the pre-procedural planning and technique used for hip aspiration candidates with suspected THA PJI. Technical factors associated with higher dry tap occurrence could possibly be avoided, whereas predictive patient-specific factors could allow clinicians to better counsel patients likely to have a dry tap.

Therefore, the primary objective of this study was to identify and evaluate the effects of various procedural and patient-specific surgical and non-surgical risk factors, as previously mentioned, on image-guided aspiration yield or dry tap occurrence at our institution in patients with clinically suspected PJI following THA.

## Methods

After receiving approval from our institutional review board, a retrospective review was conducted at a large academic orthopedic specialty hospital examining a consecutive cohort of THA patients who received image-guided hip joint aspiration procedures for diagnostic work up of suspected PJI over a 4-year period (May 2016–February 2020). Diagnostic criteria for suspected PJI were patients with elevated ESR and CRP according to the 2018 MSIS definition of PJI [26]. Cohorts were separated into two groups based on whether a dry tap occurred or not. Dry taps were defined by the presence of a saline lavage procedure in cases which little-to-no native fluid aspirate (less than 0.5 cc) for sufficient cell count or culture sample was obtained from the initial aspiration attempt as documented in the radiologist’s procedural note. Exclusion criteria included any patients less than 18 year old, absence of a THA, patients who received non-articular hip aspirations, or had an antibiotic spacer identified on pre-procedural imaging.

As the primary outcome of our study was to evaluate the effect of potential factors predicting the occurrence of a dry tap, both procedural and patient factors were identified and manually recorded via query of our institution’s electronic medical record (Epic Caboodle, version 15; Verona, WI) using Microsoft SQL Server Management Studio 2017 (Redmond, WA, USA).

The procedural factors that were collected included the type of imaging modality used for aspiration [i.e., computed tomography (CT) scan, fluoroscopy (FLU), ultrasound (US)], anatomic approach (i.e., anterior, lateral, posterior), needle gauge size used (i.e., 18, 20, 22), and the presence of a trainee (i.e., MSK radiology fellow). Imaging modality was typically under ultrasound guidance unless specified for fluoroscopy from the referring physician or if the patient body habitus would likely preclude adequate visualization of the hip with ultrasound. Our institution has extensive experience with MSK ultrasound, which is a key reason the vast majority of aspirations were performed under ultrasound guidance. CT scans were performed in circumstances when ultrasound or fluoroscopy was not possible or resources were not available.

Patient predictive factors included both surgical and non-surgical characteristics such as number of prior ipsilateral hip surgeries, femoral head size, as indicated in the operative report, ESR and CRP values collected within 14 days prior to aspiration, and most recent BMI recorded prior to aspiration. All data were collected and de-identified using Microsoft Excel software.

All image-guided hip joint aspiration procedures at our institution were performed by an attending MSK-trained radiologist with or without a fellow in-training. The study included data from several sites, which therefore encompassed many radiologists (> 10). Moreover, all patients undergoing THA at our institution were subjected to the same standardized intraoperative and postoperative management protocols known to effectively reduce PJI rates [27, 28].

### Statistical analysis

All statistical analyses were performed using SPSS version 25 (IBM Corporation, Armonk, New York). Bivariate analysis was performed using Chi-squared, Fisher’s exact tests, and Mann–Whitney U tests to compare the mean differences between cohorts for categorical and nonparametric data. A binary logistic regression was also used to determine the likelihood of a dry tap (i.e., use of a saline lavage) in the presence of these predictive factors. A cutoff *p* value of less than 0.05 was considered to be statistically significant.

## Results

Upon initial review of the data, a total of 557 patients who underwent image-guided hip joint aspiration at our institution were identified. Two hundred and twenty-one patients were excluded due to the presence of an antibiotic spacer identified on pre-procedural imaging, absence of THA, or performance of a non-articular hip aspiration. Three hundred and thirty-six patients met our inclusion criteria.

These hip aspiration procedures mainly identified suspected chronic rather than acute infections, as the mean time from the surgery date to the hip aspiration date was 319.4 (SD = 27.6) days. One hundred and twenty hip aspirations (35.7%) resulted in a dry tap where the patients underwent a saline lavage. Tables 1 and 2 detail the mean and/or count differences in procedural and patient-specific factors between dry tap and non-dry tap cohorts.

**Table 1:** Comparison of procedural factors between dry tap and non-dry tap cohorts

Factor	Dry tap		Non-dry tap		Total count	*P* value
	%	Count	%	Count		
*Aspiration modality*						
CT-guided	60%	6	40%	4	10	0.275
FLU-guided	35.7%	40	64.3%	72	112	
US-guided	34.4%	73	65.6%	139	212	
*Needle gauge*						
18-Gauge	36.7%	62	63.3%	107	169	0.051
20-Gauge	31.8%	48	68.2%	103	151	
22-Gauge	62.5%	6	37.5%	10	16	
*Aspiration approach*						
Anterior	36%	104	64%	185	289	0.960
Lateral	27%	3	73%	8	11	
Posterior	0%	0	100%	1	1	
*Trainee*						
Trainee present	37.6%	65	62.4%	108	173	0.370
No trainee	33.7%	55	66.3%	108	163

**Table 2:** Comparison of patient-specific factors between dry tap and non-dry tap cohorts

Factor	Dry tap		Non-dry tap		*P* values	
	*N*	Mean	*N*	Mean	MW	Logistic
*Surgical factors*						
# Surgeries	120	1.57	216	1.63	0.743	0.655
Head size (mm)	51	35.4	99	34.5	0.125	0.236
*Non-surgical factors*						
BMI	113	30.87	199	29.95	0.272	0.264
BMI of US-guided aspirations only	71	30.38	127	29.95	0.609	0.499
CRP (mg/L)	59	51.48	110	67.59	0.111	0.222
ESR (mm/h)	58	55.64	110	63.70	0.286	0.214

None of the procedural factors (needle gauge, imaging modality used for aspiration, anatomic approach, or the presence of a trainee) were found to be significantly different between the two cohorts (Table 1). Similarly, there were no differences found in any surgical or non-surgical factors (number of prior ipsilateral hip surgeries, femoral head size, ESR and CRP values, and BMI) studied between the two groups. Furthermore, none of these factors conferred any greater odds of a dry tap occurring as determined by a logistic regression analysis (Table 2).

## Discussion

Synovial fluid aspiration is widely used as a standard procedure to help guide management options for patients with suspected THA PJI [1–4]. Despite the frequency of this procedure, clinicians are often unable to aspirate sufficient fluid from the affected hip [10, 11]. To our knowledge, our study is the first to examine predictive factors associated with dry tap occurrence in hip aspirations of THA patients with clinical suspicion for PJI. We found that there were no statistically significant differences found between patients with or without a dry tap with respect to any of the procedural or patient-specific surgical or non-surgical predictive factors studied.

In this study, there was no statistically significant difference in the proportion of patients who had a dry tap and those who did not with respect to procedural factors such as aspiration modality and aspiration approach. Despite the lack of statistical significance, the few aspirations performed under CT guidance had a higher incidence of dry tap, when compared to fluoroscopy and ultrasound. At our institution, the vast majority of aspirations are performed under ultrasound guidance. At many other institutions, image-guided hip aspiration may be performed primarily under fluoroscopic guidance. Though no statistically significant differences in average BMI were found between aspiration modalities, this may lead to bias in our patient cohort and possibly leaves the other modalities underpowered for detection of statistically significant differences. Depending on the operator, some proceduralists may prefer ultrasound as the presence of fluid can be directly visualized, whereas fluoroscopy utilizes anatomic landmarks to access the joint. However, others may prefer fluoroscopy to ultrasound, as accessing the posterior, dependent portion of the joint space can be easier and may increase the chance of yielding joint fluid. Under ultrasound where the approach is typically anterior, it can be difficult to access the dependent posterior aspect of the hip joint as the needle can no longer be seen, due to either the presence of hardware or poor ultrasound penetration. For this reason, we also investigated whether lateral or posterior approaches might have a lower frequency of dry taps but were likely underpowered for this analysis, as the overwhelming majority of aspirations were performed through an anterior approach. Of the few aspirations performed with a lateral or posterior approach, there was a lower rate of dry taps in these patients, although not statistically significant.

Surgical factors, such as number of prior surgeries and head size, also did not show any statistically significant differences between cohorts. The hypothesis that the degree of scarring or altered anatomy from prior surgeries might lead to difficulty accessing or visualizing the joint was the motivation for including these factors in our analysis. Of note, information regarding head size was not available for all patients, which accounts for the smaller cohort. The hypothesis that larger head size may be correlated with more fluid volume, and therefore, lower frequency of dry taps was not confirmed in this study. Based on the mean time from surgery to aspiration, this study primarily examined patients with suspected chronic PJI. Dry taps may be more likely encountered in the chronic setting due to scarring and absence of postoperative joint fluid, which would theoretically be more commonly seen in the acute setting.

Patient factors such as BMI and ESR/CRP did not yield any statistically significant differences between the two groups. BMI could theoretically increase the frequency of dry tap through two possible mechanisms: difficulty in accessing the joint due to larger distance to traverse with the needle, and particularly with ultrasound, poor visualization of the needle tip related to the required depth of ultrasound penetration. Even with a subset analysis of patients who underwent ultrasound-guided hip aspirations, no statistically significant difference was found in mean BMI between the two groups. In our practice, we generally try to perform image-guided hip aspirations in obese patients under fluoroscopy for the above reasons. Finally, pre-aspiration ESR and CRP values were not statistically different between dry tap and non-dry tap cohorts, despite being important clinical markers in the workup for possible PJI. The hypothesis that patients with higher ESR and CRP values would be less likely to have a dry tap was not confirmed in this study.

There were several limitations to this study, including its retrospective design and therefore small sample sizes for certain factors such as aspiration modality and aspiration approach. In particular, our study population is disproportionately represented by hip aspirations performed under ultrasound guidance as mentioned previously. Incomplete information in the medical record regarding surgical factors such as head size may have led to the analysis of this feature being underpowered. Also, lack of easily accessible data regarding antibiotic treatment plans and duration within the medical record made it difficult to exclude all patients that may have been treated with oral antibiotics, although all patients with antibiotic cement spacers were excluded from this study. Institutional practice in general is to stop antibiotics 2 weeks prior to aspiration, however, and therefore, this likely had minimal effect on accurate patient inclusion and exclusion.

In the case of a dry tap, clinicians often use a saline lavage followed by re-aspiration to obtain sufficient fluid for analysis. However, the accuracy of this procedure is controversial. Some studies indicate that saline lavage is feasible for the diagnosis of PJI while others question the accuracy due to the risk of sample dilution, contamination, and false positive cultures upon injection of normal saline into the puncture site [8, 10–12, 14]. Salem et al. and others have found increased sensitivity and decreased specificity for PJI among saline lavage samples compared to native fluid samples and recommend the use of saline lavage [8, 10]. In contrast, Deirmengian et al. [29] found decreased sensitivity due to saline dilution of the sample and recommend the avoidance of saline lavage.

While we cannot comment on the accuracy of saline lavage cultures, our findings contribute to the current literature regarding dry tap occurrence among hip joint aspirations. In our study and in the related literature, over a third of patients undergo dry tap aspirations that may not contribute to the diagnosis of PJI [10, 12]. Understanding predictors of dry taps could help guide pre-procedural planning and thus better prepare patients that will likely undergo an invasive dry tap aspiration. This knowledge could also optimize aspiration guidelines as well as allow clinicians to better counsel patients likely to have a dry tap. We do not suggest, however, that pre-procedural factors should preclude aspiration procedures due to reports of the rate of unsuspected PJI in patients undergoing arthroplasty to be greater than 10% [30]. Further research is needed to identify other potential factors that may be more predictive of dry taps. Additionally, we believe future studies could better determine the efficacy of saline lavage by assessing concordance between culture data obtained via saline lavage and culture data obtained via intraoperative findings.

## Conclusion

Dry taps occurred in 35.7% (120 of 336) of hip aspirations within our study population. No associations with dry tap occurrence were found among the procedural, surgical, or non-surgical factors studied. Further research is needed to determine better predictors of dry tap, which could improve the pre-procedural planning of hip aspiration candidates, optimize aspiration guidelines, as well as allow clinicians to better counsel patients likely to have a dry tap.

## Data Availability

All data were identified and manually recorded via query of our institution’s electronic medical record.

## Abbreviations

PJI: Periprosthetic joint infection
THA: Total hip arthroplasty
ESR: Erythrocyte sedimentation rate
CRP: C-reactive protein
BMI: Body mass index
CT: Computed tomography
US: Ultrasound
FLU: Fluoroscopy
